# Enzyme-Linked Immunosorbent-Assay for Deoxynivalenol (DON)

**DOI:** 10.3390/toxins3080968

**Published:** 2011-08-04

**Authors:** Fang Ji, Hua Li, Jianhong Xu, Jianrong Shi

**Affiliations:** Institute of Food Safety, Jiangsu Academy of Agriculture Science (Key Lab of Agro-Food Safety and Quality, Ministry of Agriculture; Key Lab of Food Quality and Safety of Jiangsu Province—State Key Laboratory Breeding Base), Nanjing 210014, China

**Keywords:** Deoxynivalenol (DON), immunoassay, ELISA, *Fusarium graminearum*

## Abstract

Deoxynivalenol (DON), one of the trichothecene mycotoxins, is a worldwide contaminant of wheat and barley, especially when infected by *Fusarium graminearum*, the causative agent of an epidemic wheat disease called Fusarium Head Blight. Because of the high risk of DON ingestion and the possibility of frequent exposure, it is important to develop a rapid and highly sensitive method for easy identification and quantification of DON in grain samples. In this study, we have developed an indirect competitive enzyme-linked immunosorbent assay (ELISA) to detect DON in wheat. We conjugated 3-*O*-Hemisuccinyl-DON (3HS-DON) to Bovine serum albumin (BSA) and Ovalbumin (OVA), and obtained DON-specific mice antisera. The indirect competitive ELISA revealed that the optimal concentration of mice serum and the coated antigen was 1/1600 and 1/1500, respectively. The antiserum cross-reacted with the trichothecenes 3-acetyl-DON and T-2 toxin, reaching about 55.2% and 6.3%, respectively, as compared with DON. Results showed that the assay could be performed satisfactorily using an extraction buffer containing less than 15% methanol. Recovery from DON was 82–93% in grains. The linear detection range of DON in grains was between 0.01 and 100 μg/mL.

## 1. Introduction

Mycotoxins are toxic metabolites produced by fungi and are of considerable concern as they not only cause plant diseases but are also damaging to human and animal organs when contaminated food is ingested [1,2,3]. Deoxynivalenol (DON) is a common mycotoxin produced by some *Fusarium* species such as *Fusarium graminearum* and *F. culmorum* and is often found in small grains that have been infected with these *Fusarium* species. The most common small grain disease is called Fusarium head blight (FHB), which often develops following moist environmental conditions when the head is in flower.

The importance of DON to food safety is due to the severe effect that this mycotoxin has on animal systems. In 2003, the Council for Agricultural Science and Technology estimated the annual cost due to DON contamination of human food crops in the United States to be $637 million [1]. DON can interfere with both protein and DNA synthesis in the cell. Symptoms exhibited by animals afflicted with trichothecene toxicoses include vomiting, feed refusal, diarrhea, and haemorrhaging of intestines and muscles [4]. DON has also been shown to be neurotoxic and immuno-suppressive [5]. Cell signaling pathways are activated by 1 mg DON/kg body weight, through gene induction and activation of several nitrogen-activated protein kinases. DON (≥100 ng/mL) activates hematopoetic cell kinase and double-stranded RNA-activated protein kinase, which leads to apoptosis [6]. The U.S. Food and Drug Administration (FDA) has set advisory DON levels for wheat-based foods and feeds of no more than 1 µg/g in finished human foods, 10 µg/g in poultry and ruminant feed, and 5 µg/g in other animal feeds [7]. Therefore, accurate determination of the presence of low amounts of DON is important in the surveillance of food and feed in order to maintain a high grain quality for food safety.

Fusarium trichothecenes have been classified into two structurally distinct groups, Type A and Type B, based on their oxygenation pattern [8]. Type A trichothecenes are generally more toxic than type B trichothecenes, suggesting the need to be able to detect type A from type B when analyzing a sample of grain [9,10]. Commonly used methods for the determination of DON include gas chromatography (GC), gas chromatography-mass spectrometry (GC/MS), high-pressure liquid chromatography (HPLC), and thin-layer chromatography (TLC), all of which involve considerable sample preparation, time involvement, and technical expertise [11,12,13,14,15]. The desire for low-cost, rapid, field-appropriate mycotoxin detection methods has led to several new developments [16]. These include enzyme-linked immunosorbant assay (ELISA) analysis, fluorescence polarization (FP), and non-instrumental immunoassays. Commercial kits using these technologies for DON detection in grains are available. These assays require little sample preparation, are rapid and relatively simple to execute, yet are sensitive [17,18,19,20]. Pestka *et al*. reported that ELISA could be used to analyze for DON in animal plasma and tissues without extensive clean-up [21].

To improve on these commercial kits, we wished to determine the optimal concentrations of antigen/antibody for DON detection as well as to test for the recovery of DON in artificially contaminated wheat samples. We report cross-reactivity studies with other Type B trichothecenes and show the levels of DON, and cross-reactive trichothecenes, that can accumulate in various Fusarium-infested wheat lines.

## 2. Materials and Methods

### 2.1. Materials

Six-week-old BALB/c female mice were obtained from the Experimental Animal Center of Nanjing General Hospital and guinea pigs were from the Experimental Animal Center of Jiangsu Academy of Agricultural Sciences. DON, 3-acetyl-deoxynivalenol (3Ac-DON), T-2 toxin, Bovine serum albumin (BSA), ovalbumin (OVA), Freund’s complete adjuvant, and Freund’s incomplete adjuvant were from Sigma-Aldrich (Shanghai, China). 2,2-azinodi-ethylbenzothiazoline-sulfonice acid (ABTS) was from Roche (Shanghai, China). Goat anti-rabbit-IgG was from Invitrogen China. Derivatives of DON, 3-*O*-Hemisuccinyl-DON (3HS-DON), and the coupling proteins 3-HS-DON-BSA and 3-HS-DON-OVA, were synthesized in this lab based on the method of Casale [18]. The derivatives elicit an immune response whereas the smaller, non-derivatized DON will not.

### 2.2. Immunization

Six-week-old BALB/c female mice were injected intraperitoneally with 3HS-DON-BSA for immunization. Initial intraperitoneal inoculation was 100–250 μg of the DON conjugate in 0.5 mL of Freund’s complete adjuvant (1:1/v:v). Two more injections were carried out using Freund’s incomplete adjuvant at intervals of four weeks each. Subsequent intraperitoneal booster inoculations followed at 2-week intervals consisting of 100–250 μg of conjugate in 0.5 mL of saline. Guinea pigs were injected subcutaneously with 3HS-DON-BSA. All guinea pig injections were the same as for mice, with the exception of the dosage at 250–500 μg. Emulsification of the antigen with the adjuvant was performed by an injector, whereby a syringe was used to draw up both the antigen and the adjuvant and emulsification occurred when the syringe plunger was moved up and down until the solution was well mixed.

### 2.3. Specificity

Four trichothecene mycotoxins, DON, 3Ac-DON, 15-acetyl-deoxynivalenol (15Ac-DON), and T-2 toxin, were tested for cross-reactivity (CR). We used indirect competition ELISA to determine the antibody titer and cross-reactivity. For the antibody titer, we selected the maximum dilution of antiserum when the enzymatic reaction was two times greater than the negative serum. At this antibody concentration, we added DON, 3-Ac-DON, 15-Ac-DON and T-2 toxin at different concentrations (0.5 μg/mL, 5 μg/mL, 50 μg/mL, 500 μg/mL, 1000 μg/mL, 2000 μg/mL), and the concentration of coating antigen (3-HS-DON-OVA) was 2.5 μg/mL. We used the Abraham formula [19] to express the cross reaction:, *Y* = (*S*/*Z*) × 100, where S is the concentration of standard antigen that causes 50% inhibition binding and *Z* is the concentration of antigen analogues that causes 50% inhibition binding. 

### 2.4. Indirect Competitive ELISA Assay

Immunoplates were coated with 100 μL/well 3HS-DON-OVA dilution series (CBS, pH 9.6) and incubated overnight at 4 °C. After washing three times with PBST, the immunoplates were incubated in a moist chamber for 1 h at 37 °C and washed with PBST as described above. 100 µL of DON, or the extraction of sample mixed with antisera 1:1 (v:v), was added to each well of the plates, incubated and washed as described above. Goat anti-rabbit-IgG was diluted 1:500 in PBS containing 1% OVA and 100 μL/well was added. After incubation in a moist chamber for 1 h at 37 °C, plates were washed three times with PBST and 100 µL/well of ABTS substrate was added. Subsequently, plates were incubated at 37 °C for 15 min. The reactions were stopped by adding 50 µL/well of 2 M H_2_SO_4_, and the optical densities determined at 405 nm using an ELISA reader (Thermo Multiskan MK3). 

### 2.5. Optimized Working Concentration

Working concentrations of coating antigens and antisera were optimized by using an indirect competitive ELISA format. The 96-well microtiter plate (Corning, USA) was coated with a series of concentrations of the coating antigen (3HS-DON-OVA), and a series concentration of antisera in Phosphate Buffered Solution (PBS) (137 mM NaCl, 2.7 mM KCl, 8.1 mM Na_2_HPO_4_, 1.76 mM KH_2_PO_4_, pH 7.4) was added. Starting with a concentration of 2000 μg/mL of 3HS-DON-OVA, dilutions of 1/500, 1/800, 1/1000, 1/1500, 1/2000 were made in PBS. Antisera was diluted to 1/400, 1/800, 1/1600, 1/3200, 1/6400 in PBS. Absorbance at 405 nm was determined.

### 2.6. The Effect of Methanol on Detecting DON by ELISA

Reagent grade methanol was diluted in sterile distilled water to form different concentrations (0–50%) of methanol. DON then was dissolved separately with the methanol dilution for the comparison of the effect of methanol to the ELISA. 

### 2.7. Preparation of Samples

Four g of wheat sample were extracted with 20 mL of 10% methanol (v/v) in water on a rotary shaker for 1 h, then centrifuged at 7000 rpm at room temperature for 10 min. The supernatant was used in the ELISA assays. 

### 2.8. Standard Curve

Inhibition of DON was determined by the indirect competitive ELISA method. A series of DON concentrations (0.001–1000 μg/mL) were prepared by dissolving DON in PBS containing 10% methanol. The standard curve was built using Curve Expert (version 1.38; Daniel Hyams, Starkville, MS).

### 2.9. Recovery Test

DON was added (0.5 µg/g, 10 µg/g, 200 µg/g) to non-contaminated wheat, extracted as described above, and 100 µL of supernatant was added to each well of a 3-HS-DON-OVA prepared plate.

### 2.10. Calculation of the Concentration of DON in Sample

Concentration of DON (μg/g) = *C*·*V*·(1/*M*)·*D*

*C*: concentration of DON in the immunoplate well;
*V*: volume of sample (mL);
*M*: weight of sample (g);
*D*: dilution ratio.

## 3. Results and Analysis

### 3.1. Titration of Antiserum

**Figure 1:**
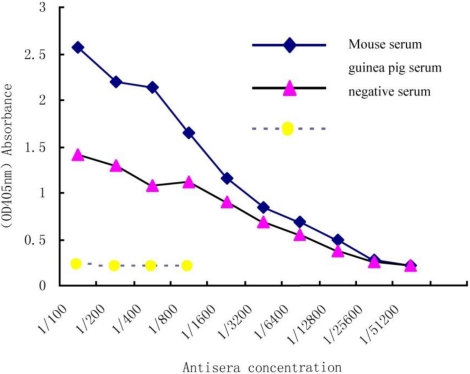
Indirect ELISA titration of Deoxynivalenol (DON), antiserum.

In order to determine an antiserum titration curve, immunoplates were coated with 3HS-DON-OVA and a series concentration of antiserum was compared to a negative serum plate. A positive value for the ELISA was defined as the absorbance value (OD_405_) of two-fold greater than the absorbance of the negative serum. As the antiserum titration curve (Figure 1) showed, the sensitivity of antibodies is clearly at 1:6400 dilutions for guinea pig serum and 1:12800 in mouse serum.

### 3.2. Optimized Working Concentration

The high titration of mouse antiserum was chosen for further experimentation. Several dilutions of the antiserum were titrated against different concentrations of the coating antigen to measure reactivity. The titer dilution of the antiserum was considered suitable when the OD_405_ value was around 1.0. We show in Table 1 that the optimized working concentration of coating-antigen and antiserum is 1:1500 and 1:1600, respectively.

**Table 1:** Determination for optimal concentrations of coated antigen and antisera.

Coated Antigen Concentration	Antisera Concentration					
	1/400	1/800	1/1600	1/3200	1/6400	
1/500	2.620	2.447	2.269	2.040	1.685	
1/500	2.590	2.421	2.231	2.006	1.654	
1/800	1.309	1.102	1.001	0.773	0.579	
1/800	1.297	1.000	0.934	0.737	0.503	
1/1000	1.187	1.093	0.964	0.770	0.513	
1/1000	1.382	1.041	0.926	0.734	0.539	
1/1500	1.448	1.222	1.059	0.884	0.629	
1/1500	1.536	1.239	1.074	0.808	0.682	
1/2000	1.508	1.210	1.124	0.900	0.648	
1/2000	1.520	1.262	1.100	0.940	0.673	

### 3.3. Effect of Methanol Concentration on the Assay

**Figure 2:**
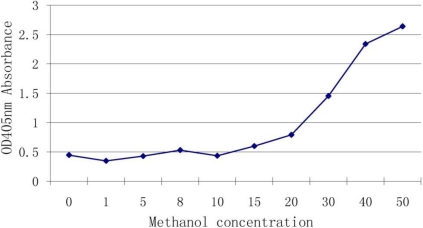
Effect of methanol concentration on the assay.

Methanol and water are often used to extract DON and we wished to determine whether the methanol can interfere with the antibody-antigen reaction. We used different concentrations of methanol in a reaction with antiserum of DON. Results (Figure 2) showed that 10% (and below) of methanol had no effect on the binding but once the concentration was higher than 20%, the interference rapidly increased.

### 3.4. Standard Curve

**Figure 3:**
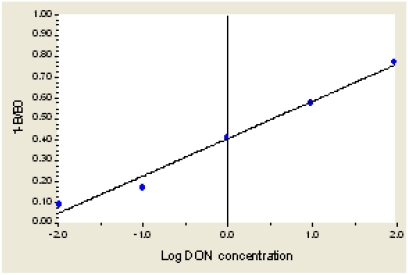
The Standard Curve for DON ELISA.

As shown in Figure 3, when the concentration of DON is 0.001 μg/mL to 1000 μg/mL, the equation of inhibition and LgC(*Y*) is *Y* = 0.3855 + 0.2075*X* + 0.0097*X*^2^ + 0.0088*X*^3^, *R*^2^ = 0.9985. When the concentration of DON is 0.01 μg/mL to 100 μg/mL, the linear equation is *Y* = 0.4057 + 0.1771*X*, *R*^2^ = 0.9915. The detection limit (IC_10_) is 0.02 μg/mL.

### 3.5. Recovery Test

To determine the precision of the DON ELISA reaction in simulated grain conditions, we added different concentrations of DON in non-contaminated clean wheat, and performed ELISA tests using our optimized conditions of 10% methanol extraction and 1/1500 coated antigen plates. Under our conditions, the ELISA technique showed (Table 2) a recovery of DON from 82% to 93%.

**Table 2:** ELISA recovery of DON from artificially contaminated wheat sample.

Added DON (μg/g)	DON Detected (μg/g)	Recovery
0.5	0.43 ± 0.02	86%
10	9.3 ± 1.1	93%
200	164 ± 35	82%

### 3.6. Specificity

In order to determine the cross-reactivity of Type A and Type B trichothecenes to 3-HS-DON-BSA, we tested a series of concentrations of each trichothecene. Results showed that the IC_50_ (half-maximal inhibitory concentration) of DON was 6.3 µg/mL, for 3Ac-DON it was 114 µg/mL, for T2-toxin (a Type A trichothecene) it was higher than 1000 µg/mL, and for 15Ac-DON it was >1000 µg/mL.

### 3.7. DON Detection in Sample

We then used our ELISA technique to measure the amount of DON found in FHB-infected wheat samples isolated from four different varieties of wheat. 

One of the varieties, Ningmai6, was susceptible to FHB while Yangmai 158 showed partial resistance, and two, Sumai3 and Wangshuibai, showed moderate to high resistance to FHB. The amount of DON present in the samples (Table 3), correlated with resistance, in that the better the resistance to FHB, the lower the levels of DON.

**Table 3:** DON Detection in different FHB resistant wheat variety with ELISA.

Wheat Variety	FHB Resistance	DON (µg/g)
Sumai3	Resistant	3.98
Wang shuibai	Resistant	2.5
Yangmai158	Middle	39.8
Ningmai6	Susceptible	63.1

## 4. Discussion

### 4.1. Emulsification

To reduce the immunogenic response in the inoculated animal, the antigen was emulsified well with adjuvant. Emulsification can be done using two possible methods, one is emulsification with a mortar and pestle, while the other one is emulsification with an injector. Although the former is easier to do, significant amounts of antigen, as much as 60–70%, may be bound and this process may also cause contamination with many microbes [22], so this method is not suitable for rare antigens. We chose the injector emulsification technique, as it produced a more stable solution that did not result in a significant loss of the antigen.

### 4.2. Immune Animal and Method

To obtain high titration and high specificity of the antibody, it is necessary to have a good antigen and an animal capable of a suitable immune response. In this study, we selected BALB/c mice and guinea pigs as the immune animals. We found the titers of antiserum from BALB/c mice to be higher than that from guinea pigs. It is not sure why this happened. Chen *et al.* (1998) suggested that an injection intradermally at multiple sites on the back was preferable [23]. When we tried this method in this study, we found it produced many sores on the animals’ back after injection and deduced this may have disturbed the production of antibody. Additionally, we found that intraperitoneal booster inoculations were much easier than the intradermal method.

### 4.3. Cross-Reactivity

Cross-reactivity (CR) to other trichothecenes is a concern when field grains are to be tested for DON. The antibody we obtained did not bind 15Ac-DON and cross-reacted with 3Ac-DON at a 55.2% level. Both 3Ac-DON and 15Ac-DON, as well as DON, are often detected in FHB-contaminated grains, but at low levels [24,25]. Indeed, detection of both acetylated versions of DON in a grain sample would be beneficial, as Alexander *et al.* [26] have shown that the acetylated versions of DON are precursors to the end-product of DON.

Our CR results differ from that of Casale *et al.* [18] (1988) who reported that their antibody to 3-HS-DON-OA actually had a higher affinity to 3Ac-DON than to DON. Schneider *et al.* [16] summarized several reports to show that 3Ac-DON generally has a stronger reaction than 15Ac-DON. This indicates that an antigen may induce antibodies that react in slightly different ways. Our result, as well as those from others, show very low cross-reaction of T-2 toxin, a type A trichothecene, with DON-induced antibodies.

### 4.4. Extraction of DON

The extraction and detection of DON in a grain sample should be as easy as possible. Solvents containing water can easily extract the water-soluble trichothecene DON. We found that the solvent containing 10% methanol and 90% water gave the best recovery of toxin. 

### 4.5. FHB-Resistant Wheat Cultivars

Resistance in wheat to FHB is not well understood but resistance in Sumai3 may be correlated with a C-3-glycosyl transferase [27]. The addition of an acetyl group to the C3 position of a trichothecene has been shown to reduce toxicity [28]. If resistant wheat lines are less susceptible to *Fusarium* infection, one would expect less visible disease symptoms and less DON production. Any DON produced may also be acetylated at the C-3 position and may not be detected in this ELISA assay. However, our results do show the correlation of reduced DON with reduced disease.

## 5. Conclusions

In this study, we made 3HS-DON-BSA conjugated DON antigen and injected BALB/c mice and guinea pigs by two different immunization methods and obtained high titers of antiserum to DON. In our system, the optimized working concentrations were 1:1500 of coating-antigen to 1:1600 of antiserum. Concentration of methanol for DON extraction of lower than 15% had little effect on the reaction. In this indirect ELISA assay, the percentage of CR of 3-Ac-DON and T-2 toxin was 55.2% and 6.3% respectively and the limit of detection was 0.01–100 μg/mL. Average recovery of DON from contaminated grain was 82%–93%. Using this method, we detected DON in field samples of wheat and found that the amount of DON correlated with the resistance to FHB.
